# Why do they take the risk? A systematic review of the qualitative literature on informal sector abortions in settings where abortion is legal

**DOI:** 10.1186/s12905-019-0751-0

**Published:** 2019-04-08

**Authors:** Sonia Chemlal, Giuliano Russo

**Affiliations:** 1 Faculty of Epidemiology and Population Health, London School of Hygiene and Tropical Medicine, London, UK; 2 Centre for Primary Care and Public Health, Queen Mary University of London, Yvonne Carter Building, 58 Turner street, London, E1 2AB UK

**Keywords:** Unsafe abortion, Legal abortion, Informal sector abortion, women’s rights, Systematic reviews, Qualitative research

## Abstract

**Background:**

Restrictive abortion laws are the single most important determinant of unsafe abortion, a major, yet preventable, global health issue. While reviews have been conducted on the extent of the phenomenon, no study has so far analysed the evidence of why women turn to informal sector providers when legal alternatives are available. This work provides a systematic review of the qualitative literature on informal sector abortion in setting where abortion is legal.

**Methods:**

We used the PRISMA guidelines to search Pubmed, Web of Science, Sciencedirect and Google Scholar databases between January and February 2018. 2794 documents in English and French were screened for eligibility against pre-determined inclusion and exclusion criteria. Articles investigating women’s reasons for aborting in the informal sector in settings where abortion is legal were included. In total, sixteen articles were identified as eligible for this review. Findings were reported following the PRISMA guidelines.

**Results:**

The review highlights the diverse reasons women turn to the informal sector, as abortions outside of legal health facilities were reported to be a widespread and normalised practice in countries where legal abortion is provided. Women cited a range of reasons for aborting in the informal sector; these included fear of mistreatment by staff, long waiting lists, high costs, inability to fulfil regulations, privacy concerns and lack of awareness about the legality of abortion or where to procure a safe and legal abortion. Not only was unsafe abortion spoken of in terms of medical and physical safety, but also in terms of social and economic security.

**Conclusion:**

The use of informal sector abortions (ISAs) is a widespread and normalised practice in many countries despite the liberalisation of abortion laws. Although ISAs are not inherently unsafe, the practice in a setting where it is illegal will increase the likelihood that women will not be given the necessary information, or that they will be punished. This study brings to the fore the diverse reasons why women opt to abort outside formal healthcare settings and their issues with provision of abortion services in legal contexts, providing an evidence base for future research and policies.

**Electronic supplementary material:**

The online version of this article (10.1186/s12905-019-0751-0) contains supplementary material, which is available to authorized users.

## Background

Unsafe abortion is defined by the World Health Organisation as any termination of pregnancy that is done either by a person “lacking the necessary skills or in an environment that does not conform to minimum medical standards, or both” [1]. The phenomenon is a significant public health issue around the world, and a major contributor to mortality in women of reproductive age. Around 8% of all maternal deaths have been attributed to unsafe abortions [2], and the figure rises to 20% in countries where abortion is prohibited [3] . Post abortion complications of unsafe abortion can be lengthy and can lead to delays in patient’s recovery, which increases risks of inappropriate treatment if a lay or formal provider needs to complete that abortion [4]. Evidence also exists that taking ineffective medication or even effective medication that is in the wrong dosage, may not cause morbidity but can be ineffective and lead to further delays and expense [5]. When administered in accordance to the WHO guidelines, pregnancy termination is one of the safest existing medical procedures [6], as complication rates have been estimated to occur only in around 1 per 1000 abortions [7]. Yet, when performed unsafely, the procedure carries a high risk of complications such as haemorrhage, renal failure, infertility and trauma to the bowel [7].

Significant differences exist in the way abortion is regulated across the world [8]. In most cases, unsafe abortions are more common in areas with more restrictive abortion legislation, such as Sub Saharan Africa and Latin America [8]. In Africa, for example, the estimated mortality due to unsafe abortion in 2008 was highest in East and Western Africa at 13,000 and 9700 respectively, where abortion laws are most restrictive [1]. Southern Africa, home to some of the countries with the most liberal abortion laws such as South Africa, Mozambique and Zambia, had the lowest rates at 500 [1]. Abortions performed outside of facilities can also have legal consequences for women and providers in most countries [9].

Although increased access to new drugs and technology such as misoprostol and mifepristone has made abortion less risky in the informal sector [10], what is often overlooked in public discourse is that access to information is often an issue in low-income settings, and, unsafe abortions, in particular those carried out in the informal sector, continue to burden women, families and societies in many countries where abortion is not legally restricted. Despite having one of the most liberal abortion laws in Africa, women in Zambia still take substantial risks to terminate unwanted pregnancies [11]. In Kenya and Tanzania policies inspired by past colonial abortion laws, still hamper access to services in the public health care service [12]. In India abortion has been legal for decades; yet the vast majority of abortions are conducted in the informal sector [3]; although informal sector abortions are not inherently unsafe, self-induced abortion and over the counter abortion drugs can be problematic if not enough or incorrect information is provided to women. In Ethiopia, despite the liberalisation of the abortion law in 2005, many terminations still take place outside health facilities [13]. In the run up to Irish abortion referendum the topic of informal abortion practices has made the headlines once again, as it emerged that informal practices and visits to abortion clinics in neighbouring countries have been common for those women denied safe and legal abortion services in their own country [14].

Although the epidemiology of the phenomenon has attracted the interest of academic scholars [15, 16] in the past, little is known about the reasons behind women’s decisions to seek informal abortion services in circumstances where such services are legally available through the formal healthcare sector. This paper sets out to systematically review the qualitative evidence on the use of informal services in countries where abortion is legal, with the aim of understanding women’s motivations behind such choice, and provide an evidence base for policies to regulate this potentially unsafe practice.

## Methods

To distinguish between countries where abortion is legal and those where it is not, we adopted the definition by The Center for Reproductive Rights [17] which categorises countries and states into four groups reflecting to their abortion law status: (a) Abortion is permitted either to save a woman’s life or outlawed altogether. Chile and El Salvador are the only two countries where abortion is completely prohibited; (b) Abortion is permitted when it is necessitated to preserve the health of the pregnant woman. Some countries in this category also permit abortion on grounds of preserving the mothers’ mental health; (c) Abortion is permitted on socioeconomic grounds, and; (d) Abortion is available on the request of the mother without restriction as to reason. In this distinction as the categories move up, the grounds on which abortion is permitted become less restrictive.

Women wishing to terminate and an unwanted pregnancy may wish to do so in a legal, formal health facility or in the informal sector either through self-inducing or visiting one of many informal sector providers, such as traditional birth attendants. The literature shows that women’s paths to seeking an informal sector abortion is complex: multiple factors, motivations and concerns come to play in the decision-making process [18–22].

**Fig. 1:**
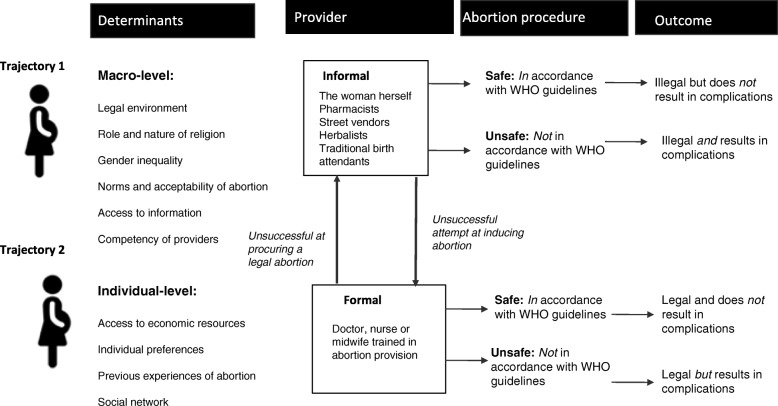
Informal sector abortion: A conceptual framework

The present review understands that women seeking informal sector abortions may do so via one of two trajectories; those who undertake the first trajectory turn to the informal sector as their first point of call for a number of reasons, such as privacy concerns, high costs and lack of awareness of abortion laws (Fig. 1). Women who undergo the second trajectory first attempt to seek a legal abortion, through a formal provider but face barriers and are forced to turn to the informal sector. Both safe and unsafe outcomes are possible; if the informal sector abortion provider carries out the abortion in accordance to the WHO guidelines [1] then despite the illegality of the procedure the outcome will be a safe abortion with minimal medical risk.

The central question addressed in this review asks: What are the reasons women who live in setting where abortion is legal choose to have an informal sector abortion? The process of this review was guided by the following research objectives [1]: To identify all the primary, qualitative literature on the reasons why women who live in settings with liberal abortion laws opt to have an informal sector abortion [2]; To extract, analyse and synthesise the relevant data on why women living in countries with liberal abortions laws end up having unsafe abortions in the informal sector [3]; To provide an up to date, global review on the reasons women in countries with liberal abortion laws opt to have unsafe abortions in the informal sector, and [4]; To increase understanding on the barriers to accessing safe, legal abortion in countries where abortion is legal.

This review focuses on qualitative research as quantitative reviews have already explored [16, 23] the impact of unsafe abortion, but in comparison, no review of the qualitative literature on this issue has been carried out. Qualitative research can be a useful tool for gaining a deep understanding of the phenomena of Informal Sector Abortion (ISA) through generating rich data on the meanings that women attach to their abortion seeking experience [24], and qualitative research is increasingly being recognised as having an important contribution to make to evidence-based healthcare and in addressing policy related questions. The Cochrane database of systematic reviews [25] and the Campbell library of systematic reviews [26] were thoroughly searched prior to the start of this current review, to ensure that there were no past or on-going reviews on the chosen topic of study.

### The search strategy

The PRISMA guidelines [27] were used to design the search strategy extract relevant information from the papers. The databases Scopus, Google Scholar, Pubmed, Web of Science and Sciencedirect were searched between the 30th of January 2018 and the 1st of February of the same year. These databases were specifically selected for their multidisciplinary nature. For example, Scopus focus covers a range of disciplines such as the social sciences, medicine, public health, humanities and women’s studies, all of which concern the subject of unsafe abortion. Multiple databases were included to ensure that all the relevant articles are captured.

Liberati et al. [28] recommends reporting the full electronic search for at least one database. A table has been included under Additional file 1 showing the exact search terms used for each database and results generated. The following search queries were inputted into the majority of databases:

(“Informal sector abortion” OR “illegal abortion” OR “clandestine abortion” OR “unsafe abortion”) AND (“legal abortion” or “abortion is allowed”) AND (“factors” OR “reasons” OR “motivations” OR “determinants” OR “motives”)

Synonyms such as ‘illegal’ and ‘clandestine’ and ‘motivations’ and ‘reason’ were used to increase search results generated. Many terms, such as the phrase ‘Termination of pregnancy’, were discarded after preliminary searches showed they did not generate any additional useful results. In some databases where very large numbers of search results were generated during the preliminary searches, additional limits were placed, such as restricting the search terms to the Titles, Abstracts and Keywords.

405 duplicates were identified and removed through hand searching. Articles were ordered according to alphabetical order of study titles and then articles whose titles and dates published matched were excluded. In order to account for any article titles that may have been misspelled, this step was repeated but with the articles being ordered according to their year of publication.

A pre-defined search criterion is one of the defining features of a systematic review [29] as it minimises bias by including articles on the basis of the criteria rather than the authors’ preferences or search results [29]. The inclusion and exclusion criteria against which the studies generated by the search results were assessed covered their topic, participants, settings, type of study, language and publication date (Table 1). No limit was placed on time of publication as this varied by study depending on when abortion has been legalised.

**Table 1:** Eligibility criteria

Criteria	Included	Excluded
Topic	● Unsafe, informal sector abortion	● Safe abortion ● Unsafe abortions that took place in a formal setting
Type of participant	● Women or friends or family of women who have undergone an unsafe, informal sector abortion ● Informal sector abortion providers	● Women who have not had an unsafe informal sector abortion ● Formal setting providers
Settings	● Country where abortion is legal either without restriction as to reason or permitted in order to preserve health or on socioeconomic grounds- according to the Centre for Reproductive Rights’ world abortion laws.	● Countries where abortion is only allowed to save a woman’s life or prohibited altogether
Type of publication	● Qualitative or mixed method	● Quantitative only
Type of study	● Original, primary research data ● Published literature	● Secondary data, reviews, opinion pieces and reports. ● Unpublished, grey literature
Language of publication	● English ● French	● Other than English or French
Publication date	● Any date after legalisation/liberalisation of laws- varies by paper	● Papers published before abortion laws were liberalised

The records generated were examined for relevance in three stages. In the first, titles were assessed against the inclusion and exclusion criteria by one of the authors (SC), allowing for the rapid elimination of ineligible articles. Articles that did not give away enough information through their titles on the relevance of their content were reserved for step two, where abstracts were assessed. In the final stage, the full texts of the remaining records obtained and assessed for eligibility. Articles whose content did not meet the criteria were eliminated and the reasons for their exclusion were documented (Additional file 1). Finally, relevant information was extracted and analysed by the two authors.

## Results

**Fig. 2:**
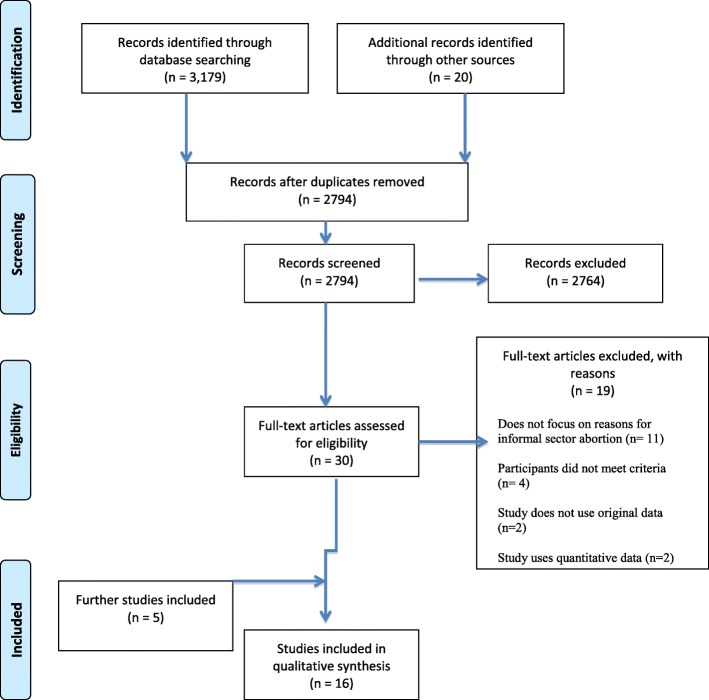
PRISMA Flow diagram of the study search

The initial search yielded a total of 3179 records were yielded, 921 of which were generated through French term searches and 2258 from English search terms. A further 20 records were identified by snowballing, that is, tracking and chasing down references in footnotes and bibliographies of the original articles and other research documents. 2764 articles were excluded after screening their titles and abstracts, leaving 30 full text articles to be assessed for their eligibility using the predefined criteria. 19 of these articles were excluded and the details on which aspect of the criteria they did not fulfil are listed under Additional file 2. A further five studies were identified through searching the reference lists of relevant articles, resulting in a total of 16 studies being included in the final synthesis (Fig. 2).

### Study characteristics

A total of 16 studies, spanning twelve countries, were included in the qualitative synthesis. The full data extraction table, complete with key study characteristics such as study type, methods, purpose and main findings, is listed in Additional file. The majority of studies were based in Sub-Saharan Africa with the exception of Northern Ireland, Great Britain, Hong Kong and the United States. The publication dates of the included studies ranged from 1998 to 2018, although the majority were published after 2010. Thirteen of the sixteen studies included recruited participants who had specified either having undergone or attempted an informal sector abortion either by recruiting women who presented at hospitals with post abortion complications or recruiting women via snowball sampling, surveys and informal sector abortion organisations such as Women on Web. Three studies focussed on members of the community who had either were ISA providers or had friends or family who had experienced an ISA (Additional file 2).

The sixteen studies covered a wide spectrum of abortion laws, from the most liberal such as South Africa and Cambodia where abortion is available on request, to countries where conditions for a legal abortion are more restricted such as Ethiopia and Kenya. Northern Ireland and the United States were outliers in this study. Northern Ireland is notorious for having some of the most restrictive abortion laws worldwide where abortion is rarely offered on legal grounds [30]. However, as its laws make a specific exception for allowing abortion in order to prevent permanent damage to a mothers physical and mental health [30], Northern Ireland was considered eligible for this review. The United States is also a unique case whereby abortion has been legal since 1973 but is regulated at state level [31]. Therefore, the extent to which abortion is restricted will vary from state to state [31].

### Women’s reasons for choosing to have an informal sector abortion

The studies surveyed widely confirmed the quantitative evidence that the practice of ISA is a widespread phenomenon: The majority of the respondents in the studies included reported being aware of women in their communities who had undergone the termination procedure clandestinely. Self-induction was found to be the preferred method of terminating a pregnancy among respondents as it was perceived to be more natural, less invasive and less medicalised [30, 32, 33], and comparable to taking contraceptive pills or painkillers [34, 35] Among participants, there was widespread knowledge of the medical risks associated with ISA. However, any fears over medical safety were outweighed by the reservations that women had about abortions in formal health facilities [20].

Ten key reasons for women opting to have an informal sector abortion, emerged in the form of themes from the literature surveyed, ranging from privacy, attitude of healthcare staff, to costs involved and timeliness of the intervention (Table 2). We review the themes below from the most to the least frequently mentioned.

**Table 2:** Reasons given to seek informal sector abortion in the literature reviewed

Source / Country	Privacy	Cost	Knowledge	Social network	Regulation	Fear of mistreatment	Unwilling staff	Self-management	Timeless-ness	Distance
Koster-Oyekani, 1998 (Zambia)	X		X		X	X				
Jewkes et al., 2005 (South Africa)	X		X	X	X	X	X		X	
Hill et al., 2009 (Ghana)	X	X	X	X						
Grossman et al., 2010 (United States)	X	X	X	X	X		X	X		
Hung, 2010 (Hong Kong)		X			X					
Rominski, Lori and Morhe, 2017 (Ghana)	X		X	X		X				
Hegde et al., 2012 (Cambodia)			X				X			
Marlow et al., 2014 (Kenya)		X	X			X	X			
Izugbar, Egesa and Okelo, 2015 (Kenya)	X				X	X				
Osur et al., 2015 (Kenya)				X						
Coast and Murray, 2016 (Zambia)	X	X	X	X	X		X			
Gerdts et al., 2017 (South Africa)	X	X	X	X		X			X	
Kebede et al., 2017 (Ethiopia)		X								
Aiken et al., 2018 (Northern Ireland)	X		X					X		X
Aiken et al., 2018 (Great Britain)	X	X	X					X	X	X
Aiken et al., 2018 (United States)	X	X			X			X		X

Concerns over *privacy* in legal health facilities were listed as a reason for women turning to informal sector providers in thirteen of the studies [11, 20, 22, 30, 32–38]. Women who chose to pursue informal sector abortion because of issues of privacy felt that the need to conceal their abortion and protect their social security outweighed their physical safety needs. Formal health facilities were deemed to be unsafe if they failed to protect a woman’s social reputation. Formal sector abortions carried the risks of being seen and kept details of the women who sought abortion meaning that the women could be easily identified as having had an abortion, whereas self-induction was preferred as it could be carried out in the privacy of one’s home [21]. The potential consequences of confidentiality breaches for women undergoing abortion are severe. Women in Great Britain, Kenya and Zambia reported fear of violence from their families, being ostracised from the community and losing their livelihoods if they were dependent on those they wished to conceal their abortion from, such as their parents or their partner [11, 33, 37].

*Costs* associated with legal abortion services was an issue that came up in ten of the studies included [11, 19–22, 32, 33, 37, 39, 40] This applied to both countries where a cost is required for formal sector abortions and in contexts where abortion is provided free of charge or covered by insurance. In countries where a fee is required, legal abortion was out of reach for women and girls from low-income backgrounds. In many cases women were dependent on the income of those who they wanted to conceal their abortion from, such as their parents or partner [11]. This also applied for when abortion is covered by insurance, as is the case in some US states, where women and girls were unable to use their insurance to acquire abortion for fear that those with whom they shared their insurance would find out about their abortion [34]. Incorrect knowledge regarding the costs of legal termination of abortion was also found to influence women’s abortion seeking behaviour. Even when abortion was freely available, such as in the case of South Africa, women held the perception that informal sector abortions would be cheaper. The perception itself of unaffordability was a barrier to accessing safe and legal abortion.

The studies reviewed also showed that a *lack of knowledge of abortion laws*, and a widespread perception that abortion is not legal, even though all of the studies included were based in contexts where abortion is permitted. Eleven of the studies included reported that one of the reasons women opted for an informal sector abortion was because they were unaware of the legality of abortion [11, 20, 22, 30, 32, 34–36, 38, 39, 41] In some cases, it was reported that this lack of knowledge was exploited by health care workers who intentionally gave the perception that abortion was not legal [39]. Some studies also pointed out that inability or confusion about how to navigate the health system compounded the general lack of knowledge of local abortion laws [42].

A review of the included studies highlighted the important role that *women’s social network* played in shaping her journey to procuring an informal sector abortion. Friends were found to be an important source of information on abortion methods and in some cases even became involved in the abortion attempt itself [20]. The role of social networks in informing women about, and leading them to informal sector abortion was documented in seven of the included studies, indicating that this was a major factor [11, 20, 22, 34, 35, 38, 43]. Knowing a public sector health worker was seen as a factor in accessing abortion services in Kenya [39]. In addition to friends, family, neighbours, teachers and even strangers whom the women had just met were also an important source of information on ISA. In Siaya, Kenya, the effect of social networks on women’s decision to have an informal sector abortion was found to be greater in younger women [43].

*Regulation* as a barrier to accessing safe, legal abortion emerged as a theme in in seven studies [11, 19, 32, 34–37] Women and girls reported the requirement of parental consent as a barrier to access, as often it was their parents that they were trying to hide the details of their abortion from. In Zambia the requirement of three doctors to approve the abortion in non-emergency cases posed issues, particularly in rural areas and regions that did not have enough doctors to meet this criterion. This was the finding of two studies; published sixteen years apart, suggesting that this has been an on-going problem for many years [11, 36]. In Hong Kong, laws that punished men who had sex with minors deterred young girls below the ages of sixteen to seek legal abortion for fear that their partners would be prosecuted [19]. In the United States where abortion regulation is decided at state level, state regulation played a major role in limiting women’s access to legal abortion [32]. For instance, in more conservative states many abortion clinics were forced to shut down, pushing women to travel longer distances for a legal abortion or turn to the informal sector [32].

Pregnant women’s’ *fears of mistreatment by staff* **-** that they will be judged, criticised, shamed and even possibly exposed - can be a deterrent for seeking a safe and legal abortion. Fear of mistreatment by staff as a reason for women choosing to avoid having legal abortions in formal health facilities was mentioned in seven studies [22, 33, 35–39] In many cases this was due to women’s past experiences or past experiences of their friends and family having been mistreated by hospital staff. Mistreatment included staff gossiping about their patients, being openly hostile, shaming the women and even accusing them of murder [35].

*Staff unwillingness to provide abortion or make a referral*. In many contexts where abortion is legal, providers may be unwilling to provide abortion for personal, religious and cultural reasons. This was a finding in five of the studies included where staff unwillingness to provide an abortion and failure to make a referral was cited as a reason for turning to the informal sector [11, 34, 35, 39, 41].

A preference for *self-managed abortions at home* was cited as a reason in four studies [30, 32–34]. Women who had sought to self-induce their abortion described a preference for their more private and familiar surroundings of their home. The home setting was considered to be less medicalised, more natural [44] and giving women a greater feeling of independence and control over their bodies. At home oral abortifacients included misoprostol and mifepristone, the standard for a medical abortion and less effective and more dangerous methods such as overdosing on vitamin c and taking herbal concoctions. The use of self-induction was also used. Where women preferred not to seek abortion from legal facilities, for fear of stigma, privacy concerns or fear of mistreatment, the use of self-induction was used to avoid the social risks and harms associated with unsafe abortion [30, 32–34].

Timeliness of services was mentioned as an additional reason in three of the studies [22, 33, 35] as *long waiting lists for regular abortion* services were identified as a key deterrent. Two of these studies were based in South Africa but published twelve years apart. This suggests that long waiting lists have been an issue affecting South African women’s chances of procuring a safe and legal abortion for many years. In Great Britain, long waiting lists often meant that women were no longer eligible for a medical abortion and would be obliged to undergo a surgical termination [33]. This reason alone was enough to put women to the informal sector.

*Distance* combined with a *lack of transport* was cited in three of the reviewed studies [30, 32, 33]. This was also closely linked to costs as the greater the distance they would have to travel for an abortion the greater the costs associated with transport. This was compounded by the fact that most abortion clinics require two or more appointments to administer the pills and follow up for complications. For women in Northern Ireland, travelling to nearby England for an abortion could pose issues for their privacy. Some women cited finding it difficult to keep private the true reason for their travels [30].

### Attributes of informal sector abortions

A key finding of this study is the existence of two pathways to seeking an informal sector abortion. In the first category, women first attempted to seek a legal abortion at a formal health facility and only turned to the informal sector when their attempts failed [11, 19, 22, 35]. Women in this trajectory were either not referred by the doctor at the clinic, not made aware of the legality of abortion or were put off by the price once they hand reached the clinic [19, 35].

The second trajectory involves women who directly sought an informal sector abortion without first trying to seek a legal service [20, 34, 38] For women in this pathway, ISA was regarded as the normal trajectory for seeking abortion. Formal sector abortion was the last resort after multiple ineffective clandestine abortion attempts or for post abortion complication treatments.

Women were aware of the medical dangers of ISA in all of the studies except one [41]. Although all of the women had sought out an informal sector abortion they were well aware of the medical risks that this form of abortion carried. All of the women interviewed by Kebede et al. [40] said they were aware of the potential complications of informal sector abortions. However, fears of physical dangers were outweighed by fears over their social safety and desperation to terminate their pregnancy [20].

One of the broad themes emerging review in this study is the great stigma that is attached to abortion, in particular if the pregnancy is the result of premarital sex. The practice of abortion could result in strong repercussions from the woman’s community and friends and family [37]. The repercussions of stigma were especially important for women who did not have an independent income as the potential loss of support of their family or partner could lose them their means of livelihood [40]. Many respondents demonstrated feelings of internalised stigma through the feelings of guilt and shame that they attached to their abortion experience [37, 40] Respondents felt that they had took part in a deviant or atypical practice [37].

The widespread use of oral abortifacients was another key theme that emerged in this review. The use of oral methods of inducing abortion, such as new drugs like misoprostol. Herbal mixtures, painkillers, hormonal preparations and household cleaning products, were the most commonly reported method of abortion in the informal sector in the majority of the included studies [11, 20, 22, 34, 35, 37, 40]. In comparison, more invasive methods such as the insertion of foreign objects [20] through the vagina or physical methods such as intensively massaging the abdomen [37] were less frequently cited.

## Discussion

To the best of our knowledge, this is the first systematic review to be conducted on the qualitative evidence around the reasons why women who live in settings where abortion is legal end up having informal sector abortions, using predominantly unsafe and ineffective methods. ISAs were reported in low-income as well as high-income countries; long waiting lists, costs, and lack of awareness about the legality of abortion appeared to be the dominant concern in the former countries, while privacy concerns and lack of insurance coverage were quoted in some high-income studies. Among the study participant’s, unsafe abortion was not spoken of solely in terms of medical and physical safety, but also in terms of social and economic security. Abortion facilities that did not protect women’s anonymity were deemed to be unsafe. This was mainly due to the fact that societal attitudes to abortion were largely negative and that severe repercussions, such as loss of livelihood and being shunned by their community, would result if it were discovered that they had procured an abortion [37]. Whilst ISA’s are not unsafe by default, in most of the studies included in this review women reported using unsafe methods such as taking herbal concoctions, using contraceptives and painkillers or inserting foreign objects into the uterus. Although some studies did report the use of a combination of mifepristone and misoprostol, which when taken appropriately can be used to safely induce an early term abortion, the methods employed to induce an abortion in the informal sector were reported to be often unsafe, suggesting that without the provision of the right information, ISAs carry an increase risk of harm to health.

A few limitations should be considered when interpreting the findings from this review. Two studies meeting our criteria could not be located, despite our best attempts to contact the authors [45, 46]. Language barriers were another restriction of our search, as only English and French articles were included, potentially introducing a language bias. A third limitation was the exclusion of grey literature, such as reports and conference abstracts, which may have introduced an element of publication bias. Furthermore, we did not search for terms such as ‘self-abortion’ and ‘self-managed abortion’, although we believe these would have been picked up by our search for ‘self-induction’ and ‘self-use’. Despite these shortcomings, we believe this review represents a meaningful contribution to the existing knowledge of why women keep taking the risk of undertaking informal abortion practices even when legal options are available. A final but important limitation is the wide heterogeneity in the abortion laws of the countries included in this review, as different countries have different laws and regulations and stipulations regulating the practice, which makes it extremely difficult to categorise countries on the basis of their abortion laws. Judging from its official regulation of the practice, Northern Ireland for example, may be considered a country with relatively little abortion restrictions, particularly considering the exceptions to protect woman’s mental health; however, women’s access to abortion services is in practice severely limited in the country.

Health workers’ competence [47] was an important factor pushing women towards the informal sector. Previous experiences of mistreatment, such as shaming and general hostility by health workers or hearing of other women’s experiences of mistreatment, was a strong deterrent to seeking a legal abortion. Some health workers were also unwilling to provide abortions or make a referral, thereby forcing women to turn to the informal sector. Studies that have investigated health workers’ willingness to provide abortion in a number of countries where abortion is legal show that overwhelmingly health workers oppose abortion and are unwilling to provide the service. As in the case of a study from South Africa reporting staff refusing to care for abortion clients, often on religious and moral grounds [48].

Women’s knowledge of abortion laws and country specific regulations was a key factor in the selection of ISA. Despite the legality of abortion and the broad grounds on which it is permitted, regulations such as the requirement for more than one doctor’s approval in areas where there is a shortage of doctors, may restrict access to legal abortion. This was evident in two studies from Zambia published sixteen years apart [11, 36]. Long waiting lists were cited as a factor by three studies, two of which are from South Africa, suggesting that is a persistent issue in South Africa. Women’s’ lack of knowledge on the legality of abortion, cited in eleven out of sixteen studies, appeared to be an important determinant of their abortion-seeking decisions. Costs were often mentioned as a barrier to access in our studies; as recent work seems to show that informal abortions can end up being as expensive as – or even more costly – than similar services in the public sector [22], it should be noted that ‘perceived costs’ could often being confused with ‘real costs’ in information-poor settings.

Whilst it is beyond the scope of this review to provide a comprehensive list of recommendations for addressing informal sector abortions, it has highlighted areas for potential intervention. For example, the significance of women’s’ social networks in influencing their abortion trajectories was an important point raised in this review. This suggests that there is potential to address the issue of ISA through education and awareness raising campaigns aimed at communities where abortion is legal. A significant proportion of unsafe abortions in the informal sector could have been averted if women’s’ social network had been excluded from the decision making process. Education campaigns could be targeted at education communities on the potential medical dangers of informal sector abortions and legal consequences in many settings where ISA’s are criminalised and could carry a prison sentence. The use of medical abortion pills for self-induction, as a harm reduction programme is another area of interest highlighted in this study. When women are provided with information on recommended dosages and administration of the drug, the evidence shows that self-induction using misoprostol can be safe [44]. Our findings regarding women’s preference for self-managed abortions for early stage pregnancies are consistent with recent studies on women’s views on the acceptability of home managed abortions [5, 49]. Governments should recognise this is rapidly becoming an option for women seeking abortion. However, the issue of cultural acceptability should be a focus for policy makers when designing healthcare services; for instance, one study carried out in the United Kingdom in 2010 found that Asian women are more likely to find self-managed abortions more acceptable than hospital based ones [49].

Future research into potential interventions should also place an emphasis on addressing long waiting lists and high costs of services which often force women to choose between having a late term abortion or turning to the informal sector. Regulations should be re-evaluated to ensure that they are reasonable in their requirements and that they do not jeopardize a woman’s right to anonymity.

## Conclusion

Unsafe abortions induced in the informal sector remain a major public health challenge in countries where abortion is legal. Following the PRISMA guidelines, we conducted a systematic review of the qualitative studies published in English and French investigating the features of the practice and the reasons behind women’s risky choice when safer alternatives are available. We screened over 2700 publications to identify a total of sixteen relevant studies exploring the practice in seven low-and middle-income countries. These studies revealed that women’s’ reasons for seeking informal sector abortions were diverse in high- and low-income settings, and their abortion seeking trajectories complex. Some themes such as the issue of long waiting lists and regulations were found to be largely country specific.

A number of gaps were identified in the literature, which may represent areas for future research on the subject. Firstly, given their importance, further primary qualitative studies appear to be needed on the phenomenon of informal sector abortion in more regions of the world where it is recognised that ISA is an issue, such as South East Asia, South America, North Africa and the Middle East. Research would also be needed on how to address the issues with formal sector abortion services identified in this study, such as privacy, affordability and timeliness. Future research on ISA should aim to understand the characteristics of women who prefer self-managed abortions over hospital-based ones.

Despite its limitations, this review shows the importance of collating and analysing systematically the information available on the drivers of unsafe abortions, and suggest future research into the safety of ISA’.

## Additional file

Additional file 1: Search strategy for the systematic literature review. Description of the methodology used to search the literature, keywords, and steps. (DOCX 23 kb)

Additional file 2: Data extraction form. Raw tabulated data and information extracted from each of the references retrieved. (DOCX 38 kb)

## Abbreviations

ISA: Informal Sector Abortion
LMIC: Low- and Middle-Income Countries
PRISMA: Preferred Reporting Items for Systematic Reviews and Meta-Analyses
WHO: World Health Organization
