# Supplementary material for: Why do they take the risk? A systematic review of the qualitative literature on informal sector abortions in settings where abortion is legal
Source: BMC Womens Health. 2019 Apr 8;19:55. doi: 10.1186/s12905-019-0751-0 (PMC6454783; doi:10.1186/s12905-019-0751-0)
Supplement: Supplementary file 1 — Search strategy for the systematic literature review. Description of the methodology used to search the literature, keywords, and steps. (DOCX 23 kb) [file 12905_2019_751_MOESM1_ESM.docx]

**Additional file 1: Search strategy**

| Date of search | Database | Search limits | Search terms | Number of results for each search | Comments |
| --- | --- | --- | --- | --- | --- |
| 30.01.18 | Scopus | TITLE-ABS-KEY  Document type- articles | ( ( "informal sector abortion" OR "illegal abortion" OR "clandestine abortion" OR "unsafe abortion" ) AND ( "abortion is allowed" OR "legal abortion" ) AND ( "factors" OR "reasons" OR "determinants" OR "motivations" OR "motives" ) )  ( reasons AND for AND illegal AND abortion ) | 126  201 |  |
|  |  |  |  | Total | 327 |
| 30.01.18 | Google scholar |  | ("informal sector abortion" OR "illegal abortion" OR "clandestine abortion" OR "informal unsafe abortion") AND ("abortion is allowed" OR "abortion is permitted") AND (“factors” OR “reasons” OR “determinants”) | 563 |  |
|  |  |  |  | Total | 563 |
| 30.01.18 | Pubmed |  | ("informal sector abortion"OR "illegal abortion" OR "clandestine abortion" OR "unsafe abortion") AND ("legal abortion" or "abortion is allowed" ) AND ("factors" OR "reasons" OR "motivations" OR "determinants" OR "motives")  Reasons for illegal abortion | 123  177 |  |
|  |  |  |  | Total | 300 |
| 30. 01.18 | Web of science | Topic, all databases | reasons for illegal abortion | 76  211 |  |
|  |  |  |  | Total | 287 |
| 30.01.18 | Science direct | All fields Sources(Social Sciences)]. | ("illegal abortion" OR "clandestine abortion" OR "unsafe abortion") and ("Factors" OR "determinants" OR "reasons" OR "motivations")[All fields Sources(Social Sciences)]. | 781 |  |
|  |  |  |  | Total | 781 |
|  |  |  | Overall total | 2258 | Previous search total was 4047 |

| Date of search | Database | Search limits | Search terms | Number of results for each search | Comments |
| --- | --- | --- | --- | --- | --- |
| 31.01.18 | Google scholar |  | ("Avortement clandestin" OR "avortement non médicalisé" OR "Avortement illégal") AND ("avortement légal" OR "l'avortement est autorisé" OR " l'avortement est légal") AND ("raisons" OR " déterminants" OR "facteurs") | 108 | Google scholar generated a lot of french results so I was able to include more search terms to limit the results |
|  |  |  | Total |  |  |
| 09.02.18 | Web of science | Title, french, articles only, all years | TITLE:("Avortement") | 400 |  |
|  |  |  | Total |  |  |
| 9.02.18 | Science direct | Title abstract key words, research articles only, limited to only french lang journals | ("Avortement") | 98 | Search results: 98 results found for TITLE-ABSTR-KEY("avortement") AND LIMIT-TO(cids, "276845,272215,272288,272219,276874,276887,276858,272150,272265,276871","Journal de Gynécologie Obstétrique et Biologi...,Gynécologie Obstétrique & Fertilité,Médecine et Maladies Infectieuses,La Revue de Médecine Interne,Annales d'Endocrinologie,La Revue Sage-Femme,Annales de Dermatologie et de Vénéréologie,Annales Françaises d'Anesthésie et de Réanim...,Comptes Rendus Biologies,Morphologie") AND LIMIT-TO(contenttype, "JL,BS","Journal"). |
|  |  |  | Total |  |  |
| 1.02.18 | Pubmed | PMC | ("avortement") AND ("raisons" OR "facteurs" OR "déterminants") | 99 | Avortement alone= 251  No results came up when I searched for avortement non médicalisé- (unsafe abortion) so I decided to keep my search broad so as to not miss out any potentially relevant articles |
|  |  |  | Total |  |  |
| 1.02.18/ | Scopus | TITLE-ABS-KEY Limit to french and English | Avortement | 216 |  |
|  |  |  |  |  |  |
|  |  |  | Overall total | 921 |  |

**Excluded studies**

| **Study** | **Country** | **Reasons for excluding** |
| --- | --- | --- |
| Harries et al., 2015 | South Africa | Study participants: Women did not have illegal abortion |
| Biddlecom et al., 2009. Guttmatcher institute | Zambia | Unpublished data + not original data |
| Smith, 2013 | Botswana | Focuses more on women's’ attitudes around abortion |
| Varkey et al., 2000 | South India | Study participants: randomly selected from village. Not selected on basis of whether they had an unsafe abortion. Also the study does not focus on informal sector abortion |
| Belton and Whittaker, 2007 | Thai border | Looks at motivations for abortion and not why women opted for an informal sector abortion |
| Baker and Khasiani, 1992 | Kenya | Focuses on unsafe abortion in general. Women who had an informal sector abortion did not mention the reasons why |
| Naveed, Shaikh and Nawaz, 2015 | Pakistan | Does not focus on informal sector abortion |
| Puri et al., 2015 | Nepal | Not all women had unsafe abortion. Focusses on women denied abortions |
| Calves, 2002 | Cameroon | Quantitative data |
| Anitete and Mayhew, 2011 | Ghana | Does not focus on reasons why women had informal sector abortion. Focuses on reasons why they chose to abortion. |
| Gursoy, 1996 | Turkey | Irrelevant to topic: focuses on history of abortion laws in Turkey |
| Visaria et al., 2004 | India | Focusses on general abortion and not informal sector abortion |
| Rossier, 2007 | Burkina Faso | Irrelevant to topic. Does not look at why women choose to have informal sector abortion but on the role of social network on the decision to have abortion. |
| Whittaker, 2002. | Thailand | Focusses on reasons why women choose to abort and not on why they choose to have informal sector abortion. |
| Bleek and Asante-Darko, 1986 | Ghana | Not original data |
| Machungo, Zanconato and Bergstrom, 1997 | Mozambique | Does not look at reasons why women choose to have an illegal abortion over a legal one.  Compares reproductive characteristics and consequences of illegal abortion. |
| Aniteye and Mayhew, 2016 | Ghana | Did not explore the reasons why women chose to opt for informal sector abortion- even though many of the women in the study had an unsafe abortion outside legal facilities. The study looked at the reasons why women choose to terminate. |
| Attindabila et al., 2014 | Ghana | Quantitative methods |
| Varga, C.A., 2002 | South Africa | Participants did not meet criteria. The study did not recruit based on whether women had an unsafe abortion or knew someone who had had an unsafe abortion. |
