# Supplementary material for: Why do they take the risk? A systematic review of the qualitative literature on informal sector abortions in settings where abortion is legal
Source: BMC Womens Health. 2019 Apr 8;19:55. doi: 10.1186/s12905-019-0751-0 (PMC6454783; doi:10.1186/s12905-019-0751-0)
Supplement: Supplementary file 2 — Data extraction form. Raw tabulated data and information extracted from each of the references retrieved. (DOCX 38 kb) [file 12905_2019_751_MOESM2_ESM.docx]

**Additional file 2: Data extraction form**

| **Author/Date** | **Country and**  **Study setting** | **Journal** | **Abortion law status** | **Study type** | **Purpose of study** | **Sample seize** | **Data collection method** | **Major findings** |
| --- | --- | --- | --- | --- | --- | --- | --- | --- |
| Aiken et al., 2018 | United States- 20 states | Perspectives on Sexual and Reproductive Health | [I](https://en.wikipedia.org/wiki/I)V | Qualitative | To find out the reasons for seeking an abortion online. To explore their experiences of ISA. | 32 women and 2 men who had sought ISA for their partner | In depth interviews | Respondents reported ordering abortion pills online.  Some respondents reported using vitamin C, black cohosh and vinegar.  Less commonly, intensive exercise, physical trauma to the stomach and inserting of objects through the vagina were reported. Some women even considered the infamous coat hanger method.  Main finding was that women encountered barriers to legal abortion.  High cost of abortion could cause a women-seeking abortion to go into debt or have to forgo paying rent.  State regulations: in more restrictive states many abortion clinics had been shut down and women had to travel long distances in order to find an abortion clinic.  Concerns about harassment due to protesters outside abortion clinics.  Lack of information on where to obtain an ISA  Many women encountered stigma when searching for a clinic, which offered abortion.  Another key finding was women’s preference for self-management, which they found to be more convenient, private and comfortable. Some women preferred to abort their pregnancies in the familiar surroundings of their home. This was regardless of whether state abortion policies were liberal or restrictive: women preferred to manage their own abortions at home.  Some women expressed a preference for a non-medical environment and a preference for independence and self-care. |
| Aiken et al., 2018 | Great Britain:  England, Scotland and Wales | Contraception | [III](https://en.wikipedia.org/wiki/I) | Mixed Method | To understand the circumstances of women seeking ISA online.  To understand why women in Great Britain might turn to ISA. | 180 women | Obtained the online consultation forms of women who had sought ISA online.  Analysed the reasons cited on the form.  Data was retrieved between November 22 2016 and March 22 2017. | The main reason women sought ISA was due to barriers to legal care.  Women experienced long waiting lists to obtain an abortion. In some cases this would have made them no longer eligible for medical abortion. Women would then have had to undergo a surgical abortion. The long waiting lists also meant that women would have to remain pregnant for longer and this caused a great deal of anxiety for many women who wanted a prompt termination in order to end their mental suffering.  Logistical reasons such as difficulty getting time off work and arranging childcare were cited.  Long distances to abortion clinics combined with a lack of transport.  Ineligibility for an NHS funded abortion was a concern mainly among undocumented migrants or women who had entered Great Britain on a visa.  Previous negative experiences with formal sector abortions dissuaded some women. It was also common for women to fear mistreatment or judgement by health workers.  Many women expressed privacy concerns concerning formal abortions. Women felt that ISA were more confidential and private. Women were desperate to ensure their privacy due to the stigma attached to abortion and out of fear that their family members might see them accessing abortion services.  Fear of violence was an important finding. Some respondents reported fearing violence from their family if they find out about their abortion and in some cases this may have resulted in loss of livelihood for women who were dependent on their family. For other women fear of violence was from a controlling partner and this also meant that in some cases they were unable to leave the house to obtain an abortion from the clinic. |
| Aiken et al., 2018 | Northern Ireland | BMJ Sexual and Reproductive Health | II | Qualitative | To understand the impact of Northern Ireland’s abortion laws on women’s decision making relating to abortion and their experiences in seeking an abortion. | 30 women | Semi-structured in depth interviews conducted between April 2017 and February 2018. | In Ireland abortion is only allowed to save a woman’s life. In Northern Ireland it is permitted to save a woman’s life and in the case of preventing “permanent damage to physical or mental health” (p.1).  The most important determinant was women not meeting the strict requirements of a legal abortion, which pushed them to the informal sector or seek abortion in nearby England.  A key finding was also that women expressed confusion about the conditions under which abortion would be legal.  Women sought ISA because they were unable to travel to England. Some women found it difficult to maintain the privacy of their abortion and conceal the true reasons for their travels if they travelled abroad for an abortion.  Costs related to obtaining childcare preventing women from travelling abroad.  For women who were not used to travelling this caused them great anxiety as in addition to travelling, they would be travelling alone and for a medical procedure that is very stigmatised.  Women expressed a preference for self-inducing due to increased feelings of independence, privacy and comfort. This was despite the legal risks that they would be taking. This was also considered more appropriate than travelling abroad and risking bleeding and pain on the journey back home.  A common finding among women who sought abortions through the web was a strong fear that police would come to them and prosecute them for self-inducing abortion.  Some respondents reported using less effective methods of self inducing such as inflicting trauma on the stomach and taking large doses of vitamin C. |
| Coast and Murray, 2016 | Zambia  Lusaka Tertiary Government hospital | Social Science and Medicine | [III](https://en.wikipedia.org/wiki/I) | Qualitative | To compare women who have safe abortions and unsafe abortions | 112 women | In Depth qualitative interviews.  Research took place between January and December 2013. | Even though legal provisions are made, women still take risks to abort and opt for clandestine abortions.  Women’s’ path for seeking abortion was complex. Many often tried multiple strategies to get a legal termination before opting for a non-clinical abortion elsewhere.  Abortion provision at government hospitals was very low. This was due to a lack of trained staff. Many trained staff were not willing to perform abortions. Insufficient doctors to meet three-opinion requirement for non emergency legal abortion.  Poor drug availability in government facilities for medical abortion was also a major issue.  Concerns over safety of abortion were outweighed by feelings of needing to conceal the abortion.  Advice respondents sought from their social network played a major role in shaping their trajectories.  Most interviewees were unaware about the legality of abortion.  Many women without their own income were dependent on those who they wished to conceal their abortion from.  For many who were able to seek a safe legal abortion this was due to chance. For example knowing someone with connections to the hospital. |
| Gerdts et al., 2017 | South Africa- Cape Town | BMC Women’s Health | [I](https://en.wikipedia.org/wiki/I)V | Descriptive | To determine the reasons for self induced abortion, methods used and sources of information about informal sector abortion | 42 Women | Face to face questionnaires. Multiple choice with yes/no.  Formative research carried out in November 2014.  Recruitment and official interviews took place in March and April 2015. | Women sought informal sector abortion because of concerns about privacy, fear of stigma and mistreatment by staff at formal facilities.  Almost all women interviewed knew of someone who had undergone an informal sector abortion.  Other community members were the main source of information about informal sector abortions.  Women’s main concerns about legal abortion services was privacy, followed by fear of mistreatment by staff. Many women feared that they would be seen at a public sector hospital by someone that knew them and that they would be found out for having an abortion.  A number of women had first attempted to have a formal sector abortion before failing and undergoing an illegal abortion.  Timelessness was a major issue.  Many women lacked knowledge on where to seek abortion.  Majority of those interviewed believed that informal sector abortions were cheaper. This was despite legal abortions being provided for free by the South African government.  Accessing informal sector abortions was described as either “Very easy” or “Somewhat easy” by most women interviewed.  Most of the abortions carried out were very unsafe with almost all women reporting heavy bleeding shortly after their illegally induced abortion. |
| Hegde et al., 2012 | Cambodia | Asia Pacific Journal of Public Health | [I](https://en.wikipedia.org/wiki/I)V | Mixed-methods | To examine the experiences of unsafe abortion by Cambodian migrant women who migrated to Thailand and returned. | 10 women interviewed.  15 took part in a survey. | Surveys, in depths interviews and document analysis.  In-depth interviews of 10 unmarried migrant women who migrated to Thailand for at least one year and returned to Cambodia. | A common finding was that women were not aware of the dangers of unsafe abortion. Women practiced abortion as a contraceptive method as they believed that contraceptive pills would cause them to become infertile.  Commonly used methods of self-inducing included abdominal massage most often combined with other methods such as drinking concoctions or Chinese abortion pulls labelled as mifepristone.  Informal sector providers in this study included midwives, market vendors and pharmacies.  One respondent described a midwife inserting sharp instruments and hot water into the vagina and using heat from a lamp.  Other women reported engaging in difficult physical work such as lifting heavy objects and taking on long work shifts.  A key finding was that women did not trust formal abortion providers to keep confidentiality. They believed that the informal sector would better ensure their confidentiality.  All respondents wrongly believed that abortion was illegal in Cambodia despite the country having some of the most liberal abortion laws in the region.  Doctors in Cambodia were very reluctant to perform abortion in unmarried women or would refer them elsewhere. Health workers believed that abortion would lead to promiscuity and encourage women to engage in premarital sex. Withholding abortion according to them would teach the women a lesson and dissuade them from engaging in premarital sex. |
| Hill, Tawiah-Agyemang and Kirkwood, 2009 | Rural Ghana- Kintampo region | Journal of Women’s Health | [II](https://en.wikipedia.org/wiki/I) | Qualitative | To explore the context of unsafe abortion in rural Ghana | 18 narratives ten focus group discussions. | Data came from eleven narratives of women who had attempted abortion and seven narratives of deaths due to abortion.  This was followed by ten focus group discussions.  The study was conducted in June 2005. | Women opted for cheaper, accessible, unsafe methods of abortion that could be kept a secret.  Social networks played an important role in directing women towards informal sector abortions.  None of the women interviewed were aware of the legality of abortion in Ghana.  Post abortion complications were usually managed at home.  Sequential abortion methods: most attempted many abortion methods starting with the cheapest and mildest. When this failed they gradually worked their way up to more dangerous methods of aborting.  Safe and legal abortion services should be the first point of call rather than the last resort after many failed attempts. |
| Izugbara, Egesa and Okelo, 2015 | Kenya  Six Kenyan public facilities specially selected for providing post abortion care. | Social Medicine and Health | [II](https://en.wikipedia.org/wiki/I) | Qualitative | To understand the social dimension of abortion safety.  To examine women’s perspectives on abortion and choice of abortion facility. | 50 women | Interviews carried out between 2012 and 2013. | Abortion is deemed unsafe if it does not protect both the woman’s social reputation and health.  Stigma was widespread. Participants felt that they had engaged in a deviant activity by procuring an abortion.  Women did not describe abortion safety only in terms of physical health but also social, reputational, relationship and economic security.  Women feared consequences if the details of their abortion were revealed. For example being shunned by community members and their family, loss of their reputation and livelihood.  Formal health facilities were deemed to be insensitive to women’s social security needs. They often required parental consent, presence of the husband and retain copies of the woman’s details. With informal sector abortions it was anonymous. Women could not be identified or traced back.  There were reports of staff gossiping, making women feel ashamed and in some cases exposing their abortion.  Hospital abortions were considered to be very costly and out of reach for the poor. |
| Jewkes et al.,2005 | South Africa  Guateng province | JOG: An International Journal of Obstetrics and Gynaecology | [I](https://en.wikipedia.org/wiki/I)V | Descriptive | To explore why women still abort outside of legal facilities.  To explore the circumstances of unsafe abortion outside of formal facilities. | 46 Women | Interviewer administered questionnaire with open and closed questions | Reasons why women opted for clandestine abortions included lack of knowledge on legality, fear of mistreatment by staff and unable to meet the legal requirements for abortion.  The most commonly cited providers were traditional healers, chemists, nurses and doctors.  Methods used commonly involved oral ingestion of products such as laxatives. Mechanical methods were only used by a small number of women.  Women reported visiting formal health facilities and not being told of the legal status of abortion or being referred to a service.  More than half of the respondents were unaware that abortion was legal in South Africa.  Some women reported previous experiences of the service, which were negative and included staff gossiping and accusing them of murder.  Social network: friends and family directed women towards informal sector abortion.  Women opted for the informal sector for a quick and private abortion. In the formal sector there were long waiting lists and women had concerns about privacy. |
| Kebede, Middelthon and Hilden, 2017 | Ethiopia- Addis Ababa | Healthcare for Women International | [II](https://en.wikipedia.org/wiki/I) | Ethnographic | To explore the routes to abortion taken by young unmarried women | 25 women residents of Addis Ababa | Participant observation, focus group discussions and interviews. Individual repeat interviews with women who had engaged in unsafe abortion were conducted to explore key issues and themes further.  Research was carried out over two periods. The first of which was December 2006 to November 2007. The second of which was December 2009 to March 2010. | Informal sector abortion was seen as being affordable and socially safe.  Legality of abortion was not an important consideration to the women interviewed.  Women weighed complex social, economic and medical issues before deciding on which abortion trajectory to pursue.  Abortion is widely condemned in Ethiopia- Especially if it is the result of sex outside of marriage. It could compromise a woman's future marriage prospects and cause a shift in her status. These concerns over social consequences of it being discovered that they had an abortion outweighed worries about the potential medical consequences of unsafe abortion/  Moral legitimacy was a key theme: many unmarried women felt it was not appropriate for them to use abortion services located in maternal and child health units. They felt that abortion services located here were not for women like them.  Women chose providers based on their location and discreteness of the service. Many women purposefully chose a geographically distant service. Public facilities were seen as difficult to protect identity- this was a major cause of concern among unmarried women. |
| Koster-Oyekani, 1998 | Zambia- Western Province:  Senanga Kaoma, Kalabo and Sesheke | Social Science and Medicine | [III](https://en.wikipedia.org/wiki/I) | Community based Mixed-Methods study | To investigate the factors influencing high levels of unwanted pregnancy in the Western Province | Questionnaire: 1273 girls  Interviews: 803 women  Focus groups: 26 in total averaging 12 participants each. The range of participants for each focus group was 9 to 18. | Self-administered questionnaires, interviews and focus groups took place during July 1994 and February 1995. | Illegally induced abortion is a major public health issue in Zambia. Legal abortion services are not accessible to most women in western province.  More than two thirds of women were unaware of the legality of abortion.  Fear of their privacy being violated or being mistreated by staff was another key reason for why women avoided formal health facility abortions.  The condition of requiring three doctors to sign the abortion papers impeded access in regions that had only one doctor. |
| Marlow et al., 2014 | Kenya  Busia, Bungoa and Trans-Nzoia counties | Reproductive Health Matters | [II](https://en.wikipedia.org/wiki/I) | Community based Mixed methods study | To explore the cultural and social norms that influence access to safe abortion | 8-14 participants in each focus group. | Focus group discussions, in depth interviews and mystery client visits  Study took place over three weeks in October 2012. | Majority of the methods women chose to abort were unsafe. The social and cultural environment is not supportive of women who have had abortions.  Methods described included ingesting medication and herbal preparations. In some cases sticks and metal were inserted into the vagina.  A number of women were unsure of the legality of abortion. Some providers intentionally used this to their advantage to mislead women into thinking abortion was illegal.  Cost at hospital or private clinic was significantly higher than most women’s income. This was a major barrier to access.  There was widespread perception that hospital methods used are not safe.  Some women feared that they would be mistreated by hospital staff. Often this fear was based on past experiences at the formal sector. |
| Osur et al., 2015 | Kenya- Siaya county | African Journal of Reproductive Health | [II](https://en.wikipedia.org/wiki/I) | Mixed methods | To examine the role of social networks in facilitating informal sector abortion | 320 women for the quantitative arm of the study.  Interviews of 15 community members.  2 focus group discussions with 8 participants in each. | Cross sectional survey, focus groups, case studies and key informant interviews.  The study took place during June 2011 and July 2012. | Almost all women interviewed said that they consulted their social networks prior to deciding on unsafe abortion. Programmes aimed at reducing unsafe abortions should be designed with this in mind.  A small number of women reported being coerced into having an informal sector abortion.  The effect of social network was greater the younger the age of the women deciding to abort.  In some cases teachers directed pregnant students towards informal sector abortion providers. Women also consulted other women who had had an abortion before. |
| Hung, 2010 | Hong kong | Reproductive Health Matters | [III](https://en.wikipedia.org/wiki/I) | Exploratory | To examine women from deprived backgrounds’ access to safe legal abortion | 29 women | Individual in depth interviews  Study took place between December 2007 and September 2008 | Many young women resort to unsafe illegal abortions or abortions in mainland china. Access to safe abortion is constrained by factors such as age, parental consent requirements and laws against sex with minors. Men who have sex with girls under the age of 16 may be imprisoned for up to five years.  Public and private hospitals in Hong Kong require parental consent for under 18’s to undergo abortion.  Cost was a major barrier, particularly for girls who come from low-income backgrounds. Safe and legal abortion was often delayed by a lack of money. By the time women had collected the money the pregnancy had advanced and the price was increased. |
| Rominski, Lori and Morhe, 2017 | Ghana- Ashanti region | Journal of Family Planning and Reproductive Healthcare | [II](https://en.wikipedia.org/wiki/I) | Qualitative | To explore why young women choose to have informal sector abortions when legal abortion is available | 29 women interviewed in addition to 8 focus groups with between 6 and 10 participants each | Interviews and focus groups based on grounded theory design were used. Questions were open ended and included probes if issues arose that the interviewer wanted to explore further. | The main themes that emerged were the role of women’s social-networks lack of knowledge about legality of abortion, fear of mistreatment by staff and concerns over privacy.  Self-induction was found to be highly normalised: it was the normal trajectory for women with unwanted pregnancies. Formal health care providers were usually only consulted in cases of complications.  There was widespread perception among women interviewed that abortion is illegal. Some women also reported being afraid of prosecution for abortion- although this was a rare occurrence in Ghana.  Many women conducted abortion at home instead of clinics because of fears of their privacy being violated and being found out for abortion.  Women's’ social network such as friends, family and neighbours were found to be the main source of information on informal sector abortion.  Many women reported past experiences of rude and hostile clinic staff. Although this is a general problem with the healthcare system it was especially important in the context of abortion due to the stigma attached to the procedure.  The author recommends making medical abortion, such as through the use of misoprostol, more easily accessible. Medical abortion is safe, private, acceptable and ideal for low income countries because of its affordability and stability at room temperature. |
| Grossman et al., 2010 | United states- Boston, San Francisco, New-York and Texas | Reproductive Health Matters | [I](https://en.wikipedia.org/wiki/I)V | Qualitative | To explore knowledge and experiences of abortion in four US cities.  To explore women in the United State’s motivations, experiences and reflections on self induced abortion and to explore the policy implications of these findings for improving access to safe and legal abortion. | 30 women | In Depth semi structured interviews carried out according to grounded theory.  Research took place between 1979 and 2008 as part of a larger study. | Self-induction viewed as natural, readily accessible, non-invasive and private whereas clinic abortion was seen as highly medicalised and invasive.  The use of Caribbean drink Malta was the most commonly used method for self-induction, followed by herbal use and physical actions such as massage or intense exercise.  All women interviewed had a negative view of clinical abortion. A number of women reported previously negative clinic experiences such as the long process of questioning and being tested for STDs. Many women were also aware of negative clinic experiences of their friends and family.  Friends and family were an important source of information regarding self-induction methods.  Most women acknowledged that clinic abortions were safer.  Women reported difficulties accessing safe abortion such as not knowing where to seek help and the requirement of parental consent- even in states where parental consent is not a requirement for legal abortion. Women also reported their physicians not appropriately directing them to where they could access abortion services.  Cost was a major factor in a woman's’ decision to abort outside legal facilities. A number of states restrict coverage for abortion by private insurance. Some women feared that if they used insurance to cover their fees their parents would find out.  Women’s reasons for choosing self-induction were the same reasons why women choose medical abortion over surgical abortion. Self-induction was viewed as more natural had less of an impact on everyday life and was compared to regulating menstruation or the emergency contraception pill. This was more in line with their religious and ethical views. |
